# Supplementary material for: Comparison of the Metabolic Profile between Unstimulated and Stimulated Saliva Samples from Pregnant Women with/without Obesity and Periodontitis
Source: J Pers Med. 2023 Jul 11;13(7):1123. doi: 10.3390/jpm13071123 (PMC10381358; doi:10.3390/jpm13071123)
Supplement: Supplementary file 1 [file jpm-13-01123-s001.zip › Supplementary file S1.pdf]

Supplementary file S1. Intergroup comparations for SS samples (univariate and multivariate analyses; and MSEA)

Figure S1(A) shows the scores plot between the first two principal components (PCs) of sPLS-DA, while Figure S1(B) shows the loadings plot of component 1 from the sPLS-DA. Table S1 shows the univariate analysis through the normalized metabolite concentration of the stimulated saliva samples shown as the mean and standard deviation. The superscript (1) after the metabolite name indicates statistical difference in the univariate analysis, (2) indicates statistical difference in the multivariate analysis (according to the loadings factor from sPLS-DA).

The scores plot between the first two principal components (PCs) of sPLS-DA indicates mainly a difference of OP and NP groups from controls (OWP and NWP), but with no great distinction between them. According to the univariate analysis, OP and NP groups showed higher concentration of butyric acid, isovaleric acid, leucine, valine, isoleucine, propionic acid, acetic acid, trimethylamine, ornithine, and phenylalanine. Besides the higher levels of propionic acid, butyric acid, and acetic acid, the OP and NP groups showed lower concentrations of proline, *N*-Acetylglutamine acid, citric acid, pyruvic acid, lactate, *N*-Acetylneuraminic acid, and galactose, according to the multivariate analysis by loading factor of component 1 from sPLS-DA.

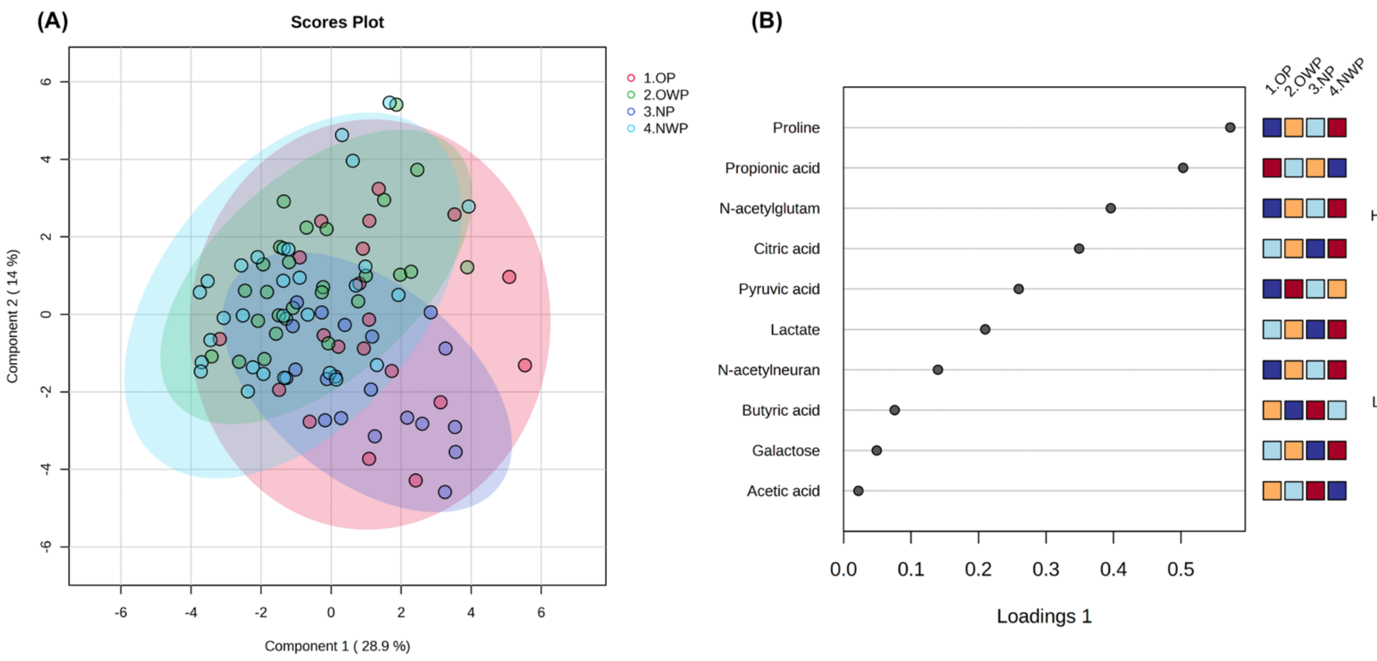

Figure S1. Scores plot between the first two principal components (PCs) of sPLS-DA **(A)**, and loading plot of component 1 from the sPLS-DA **(B)**.

Table S1. Univariate analysis of the Stimulated Saliva samples. Normalized concentration of metabolites shown as the mean and standard deviation for each group (ANOVA and Tukey test as post-hoc).

HMDB Card	Metabolites	CS (ppm)	OP (n = 20) Mean ± SD	OWP (n = 27) Mean ± SD	NP (n = 20) Mean ± SD	NWP (n = 29) Mean ± SD	p
**HMDB0000491**	3-Methyl-2-ketovaleric acid	0.85	0.41 ± 0.21	0.36 ± 0.17	0.49 ± 0.21	0.37 ± 0.18	0.152
**HMDB0000039**	Butyric acid [1,2]	0.89	0.32 ± 0.25 **AB**	0.18 ± 0.14 **A**	0.40 ± 0.23 **B**	0.18 ± 0.14 **A**	**<0.001**

**HMDB0000718**	Isovaleric acid [1]	0.91	0.27 ± 0.23 **AB**	0.15 ± 0.10 **A**	0.33 ± 0.20 **B**	0.17 ± 0.09 **A**	**<0.001**
**HMDB0000687**	Leucine [1]	0.97	0.29 ± 0.27 **A**	0.16 ± 0.09 **B**	0.27 ± 0.13 **AB**	0.17 ± 0.10 **AB**	**0.006**
**HMDB0000883**	Valine [1]	0.99	0.24 ± 0.26 **A**	0.10 ± 0.07 **B**	0.23 ± 0.15 **A**	0.10 ± 0.07 **B**	**0.001**
**HMDB0000172**	Isoleucine [1]	1.01	0.25 ± 0.27 **A**	0.10 ± 0.07 **B**	0.21 ± 0.14 **AB**	0.11 ± 0.09 **B**	**0.002**
**HMDB0000237**	Propionic acid [1,2]	1.06	0.30 ± 0.23 **A**	0.17 ± 0.15 **AB**	0.30 ± 0.20 **A**	0.15 ± 0.14 **B**	**0.004**
**HMDB0003156**	2,3-Butanediol	1.14	0.23 ± 0.19	0.18 ± 0.18	0.16 ± 0.06	0.17 ± 0.19	0.606
**HMDB0000108**	Ethanol	1.18	0.22 ± 0.22	0.20 ± 0.14	0.19 ± 0.11	0.15 ± 0.11	0.376
**HMDB0000174**	Fucose	1.25	0.36 ± 0.25	0.31 ± 0.21	0.35 ± 0.22	0.35 ± 0.20	0.817
**HMDB0003243**	Acetoin	1.38	0.27 ± 0.22	0.22 ± 0.14	0.28 ± 0.11	0.18 ± 0.10	0.077
**HMDB0000161**	Alanine	1.48	0.33 ± 0.25	0.26 ± 0.16	0.38 ± 0.20	0.32 ± 0.16	0.224
**HMDB0001414**	Putrescine	1.76	0.27 ± 0.25	0.17 ± 0.11	0.26 ± 0.20	0.20 ± 0.12	0.146
**HMDB0000042**	Acetic acid [1,2]	1.92	0.31 ± 0.22 **A**	0.20 ± 0.17 **AB**	0.32 ± 0.18 **A**	0.17 ± 0.11 **B**	**0.008**
**HMDB0000230**	*N*-Acetylneuraminic acid [2]	2.21	0.38 ± 0.27	0.31 ± 0.19	0.43 ± 0.18	0.35 ± 0.21	0.317
**HMDB0003355**	5-Aminopentoate	2.24	0.28 ± 0.24	0.21 ± 0.14	0.26 ± 0.17	0.21 ± 0.13	0.423
**HMDB0006029**	*N*-Acetylglutamine [2]	2.33	0.30 ± 0.21	0.32 ± 0.20	0.34 ± 0.19	0.39 ± 0.23	0.408
**HMDB0000243**	Pyruvic acid [2]	2.37	0.27 ± 0.21	0.29 ± 0.17	0.28 ± 0.13	0.29 ± 0.15	0.959
**HMDB0000254**	Succinic acid	2.41	0.35 ± 0.26	0.30 ± 0.19	0.34 ± 0.25	0.32 ± 0.20	0.869
**HMDB0000094**	Citric acid [2]	2.53	0.29 ± 0.21	0.28 ± 0.14	0.28 ± 0.13	0.36 ± 0.22	0.288
**HMDB0000164**	Methylamine	2.60	0.24 ± 0.24	0.15 ± 0.08	0.22 ± 0.10	0.17 ± 0.07	0.059
**HMDB0000906**	Trimethylamine [1]	2.89	0.14 ± 0.14 **AB**	0.06 ± 0.03 **A**	0.21 ± 0.20 **B**	0.07 ± 0.03 **A**	**<0.001**
**HMDB0000214**	Ornithine	3.06	0.30 ± 0.25 **A**	0.15 ± 0.09 **B**	0.25 ± 0.19 **AB**	0.19 ± 0.11 **AB**	**0.021**
**HMDB0000097**	Choline	3.20	0.24 ± 0.26	0.18 ± 0.14	0.29 ± 0.15	0.19 ± 0.11	0.134
**HMDB0000904**	Citrulline	3.14	0.13 ± 0.21	0.07 ± 0.04	0.10 ± 0.03	0.07 ± 0.03	0.119
**HMDB0004983**	Dimethylsulfone	3.15	0.30 ± 0.26	0.23 ± 0.17	0.28 ± 0.12	0.23 ± 0.11	0.391
**HMDB0001875**	Methanol [1]	3.36	0.28 ± 0.23 **AB**	0.16 ± 0.11 **A**	0.23 ± 0.13 **AB**	0.28 ± 0.18 **B**	**0.038**
**HMDB0000251**	Taurine	3.43	0.25 ± 0.22	0.16 ± 0.09	0.22 ± 0.08	0.18 ± 0.09	0.088
**HMDB0000123**	Glycine	3.56	0.28 ± 0.23	0.21 ± 0.14	0.21 ± 0.13	0.23 ± 0.15	0.521
**HMDB0000190**	Lactate [2]	4.12	0.20 ± 0.25	0.19 ± 0.12	0.16 ± 0.08	0.19 ± 0.11	0.794
**HMDB0000162**	Proline [2]	4.14	0.30 ± 0.23	0.30 ± 0.17	0.30 ± 0.12	0.35 ± 0.19	0.710
**HMDB0000122**	Glucose	5.24	0.16 ± 0.26	0.09 ± 0.12	0.11 ± 0.13	0.11 ± 0.15	0.655
**HMDB0000143**	Galactose [2]	5.27	0.34 ± 0.21	0.32 ± 0.23	0.27 ± 0.14	0.34 ± 0.21	0.683
**HMDB0000163**	Maltose	5.41	0.09 ± 0.22	0.08 ± 0.17	0.04 ± 0.04	0.07 ± 0.12	0.787
**HMDB0000294**	Urea	5.78	0.32 ± 0.22	0.33 ± 0.17	0.33 ± 0.23	0.33 ± 0.17	0.998
**HMDB0000296**	Uridine	5.91	0.30 ± 0.23	0.30 ± 0.20	0.29 ± 0.22	0.26 ± 0.12	0.888
**HMDB0000134**	Fumaric acid	6.52	0.50 ± 0.19	0.48 ± 0.12	0.46 ± 0.13	0.49 ± 0.13	0.879
**HMDB0000020**	para-Hydroxyphenylacetic acid	6.87	0.34 ± 0.21	0.29 ± 0.19	0.38 ± 0.17	0.28 ± 0.19	0.370
**HMDB0000158**	Tyrosine	6.90	0.29 ± 0.24	0.20 ± 0.13	0.26 ± 0.08	0.21 ± 0.14	0.165
**HMDB0000177**	Histidine	7.07	0.30 ± 0.21	0.23 ± 0.16	0.26 ± 0.11	0.24 ± 0.16	0.584
**HMDB0000159**	Phenylalanine [1]	7.43	0.31 ± 0.25 **A**	0.16 ± 0.10 **B**	0.27 ± 0.11 **AB**	0.18 ± 0.13 **B**	**0.004**
**HMDB0000142**	Formic acid	8.45	0.16 ± 0.15	0.18 ± 0.23	0.13 ± 0.11	0.10 ± 0.15	0.418

HMDB, human metabolome database; CS, chemical shift, OP, obesity and periodontitis; OWP, obesity without periodontitis; NP, normal BMI with periodontitis; NWP, normal BMI without periodontitis; SD, standard deviation; *p*, significance level; [1] univariate analysis (ANOVA with post-hoc Tukey); [2] multivariate analysis using the loading factor of component 1 from sPLS-DA. Different bold letters mean statistically significant differences between groups. Bold values, statistical significance lower than 5%.

Metabolite set enrichment analysis (MSEA) was conducted to identify patterns of metabolite concentration changes considering the metabolite pathway and subclasses of metabolites. Thus, we described the MSEA for visualization and functional analysis of metabolite data. Metabolites that were in higher concentrations according to the univariate and multivariate analyses in the intergroup comparations of SS samples (butyric acid, isovaleric acid, leucine, valine, isoleucine, propionic acid, acetic acid, trimethylamine, ornithine, and phenylalanine) were included (Figure S2). The three top pathways were: Valine, leucine and isoleucine degradation; Fatty acid biosynthesis; and Propanoate metabolism (Figure S2-A). The main metabolic subclasses were: Amino acids; Saturated fatty acids; and Straight chain fatty acid (Figure S2-B). In contrast, metabolites that were in lower concentrations according to the multivariate analysis in the intergroup comparations of SS samples (Proline, *N*-Acetylglutamine acid, citric acid, pyruvic acid, lactate, N-Acetylneuraminic acid, and galactose) were also included in the MSEA (Figure S3). The five top pathways were: Warburg effect; Transfer of acetyl groups into mitochondria; Citric acid cycle; Amino sugar metabolism; and Gluconeogenesis (Figure S3-A). The main metabolic subclasses were: Amino acids; *N*-Acylneuraminic acids; and Short-chain acids and derivatives (Figure S3-B).

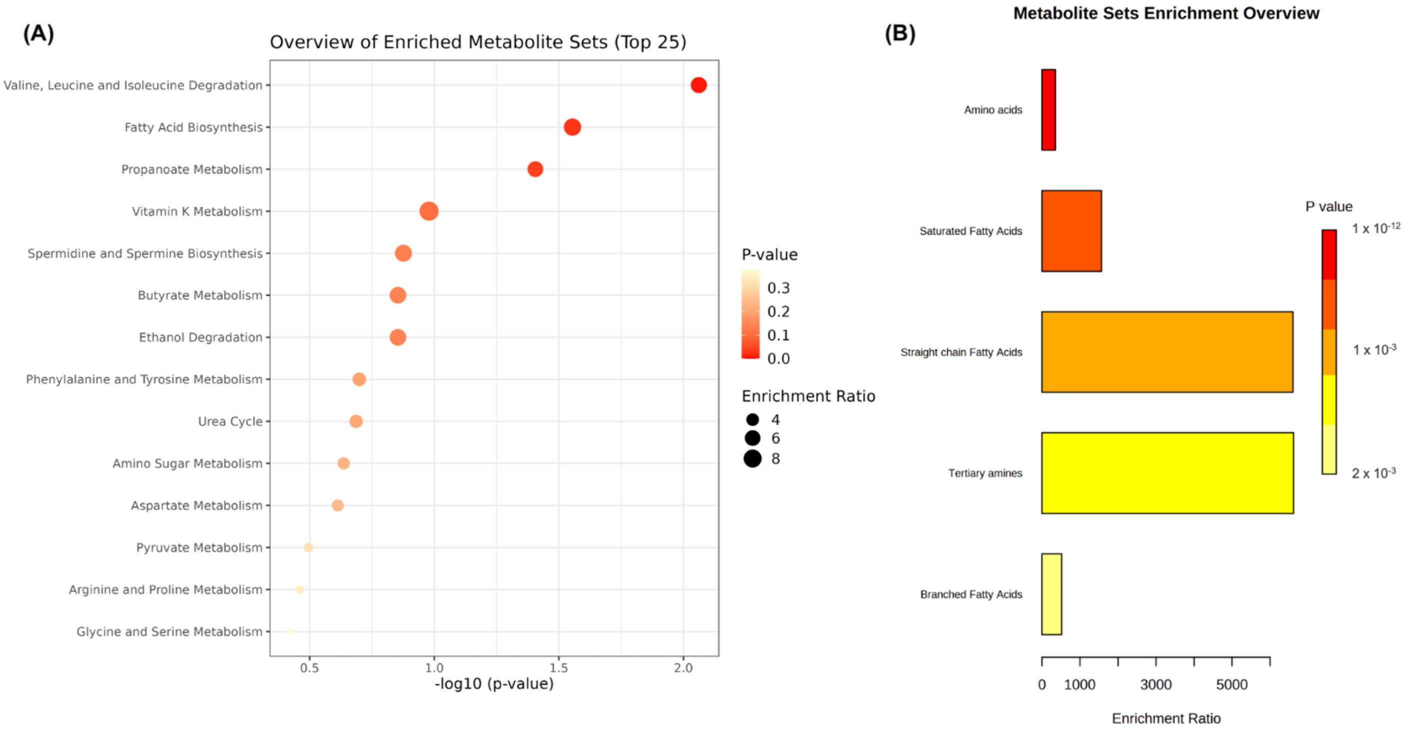

Figure S2. Main pathways (A) and subclasses (B) of higher concentrated metabolites in stimulated saliva samples from OP and NP groups assigned by MSEA.

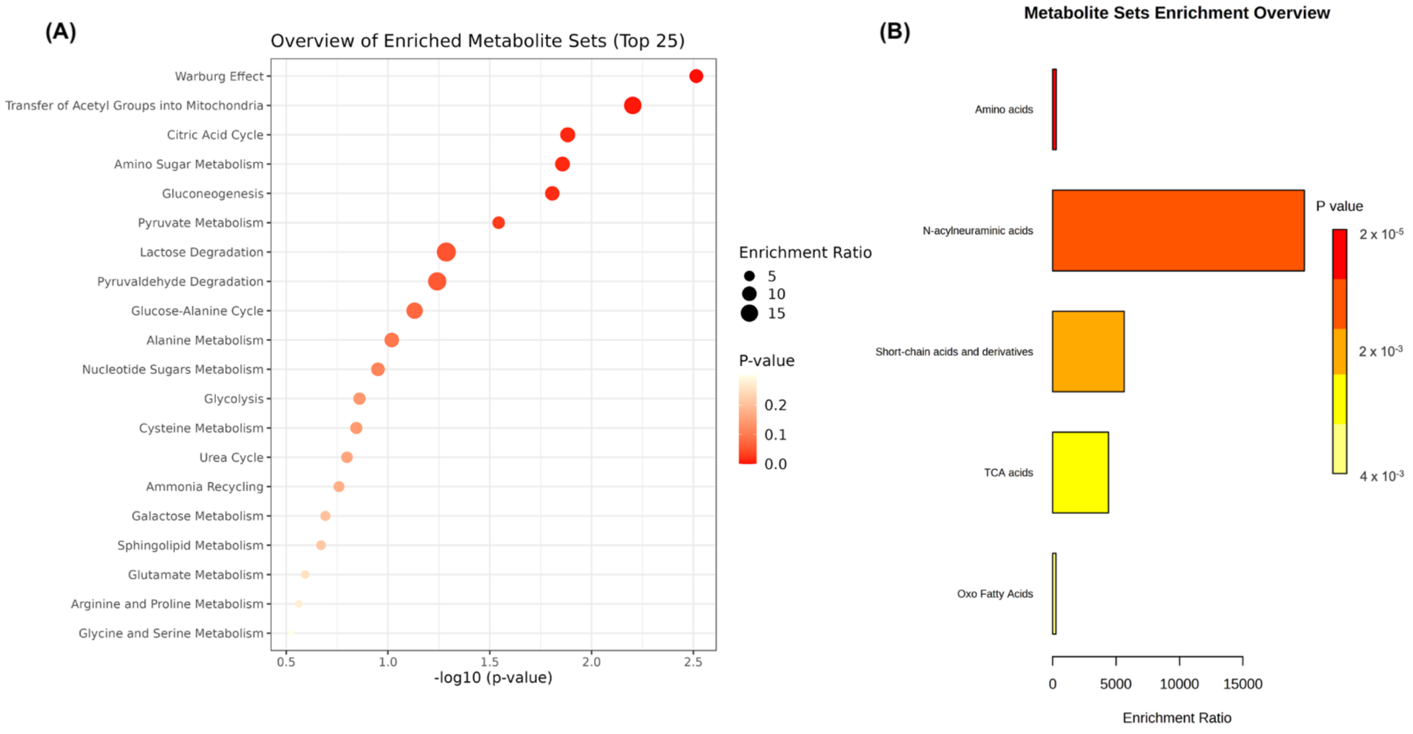

Figure S3. Main pathways (A) and subclasses (B) of lower concentrated metabolites in stimulated saliva samples from OP and NP groups assigned by MSEA.
