# Supplementary material for: Comparison of the Metabolic Profile between Unstimulated and Stimulated Saliva Samples from Pregnant Women with/without Obesity and Periodontitis
Source: J Pers Med. 2023 Jul 11;13(7):1123. doi: 10.3390/jpm13071123 (PMC10381358; doi:10.3390/jpm13071123)
Supplement: Supplementary file 1 [file jpm-13-01123-s001.zip › Supplementary file S2.pdf]

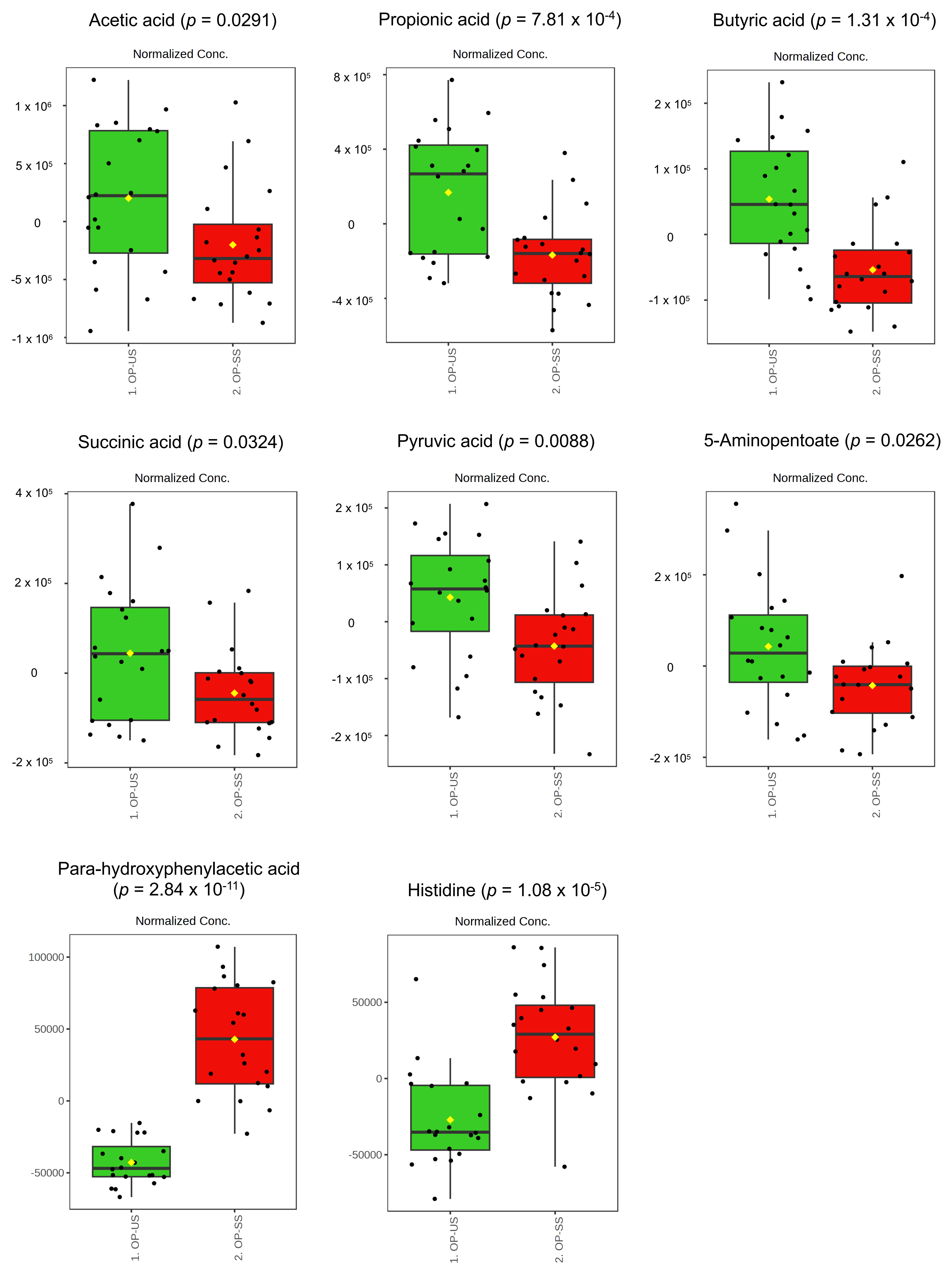

Acetic acid (p = 0.0291)
Normalized Conc.
1 x 10^6
5 x 10^5
0
-5 x 10^5
-1 x 10^6
1. OP-US
2. OP-SS
Propionic acid (p = 7.81 x 10^-4)
Normalized Conc.
8 x 10^5
4 x 10^5
0
-4 x 10^5
1. OP-US
2. OP-SS
Butyric acid (p = 1.31 x 10^-4)
Normalized Conc.
2 x 10^5
1 x 10^5
0
-1 x 10^5
1. OP-US
2. OP-SS
Succinic acid (p = 0.0324)
Normalized Conc.
4 x 10^5
2 x 10^5
0
-2 x 10^5
1. OP-US
2. OP-SS
Pyruvic acid (p = 0.0088)
Normalized Conc.
2 x 10^5
1 x 10^5
0
-1 x 10^5
-2 x 10^5
1. OP-US
2. OP-SS
5-Aminopentoate (p = 0.0262)
Normalized Conc.
2 x 10^5
0
-2 x 10^5
1. OP-US
2. OP-SS
Para-hydroxyphenylacetic acid
(p = 2.84 x 10^-11)
Normalized Conc.
100000
50000
0
-50000
1. OP-US
2. OP-SS
Histidine (p = 1.08 x 10^-5)
Normalized Conc.
50000
0
-50000
1. OP-US
2. OP-SS
