# Supplementary material for: Comparison of the Metabolic Profile between Unstimulated and Stimulated Saliva Samples from Pregnant Women with/without Obesity and Periodontitis
Source: J Pers Med. 2023 Jul 11;13(7):1123. doi: 10.3390/jpm13071123 (PMC10381358; doi:10.3390/jpm13071123)
Supplement: Supplementary file 1 [file jpm-13-01123-s001.zip › Supplementary file S3.pdf]

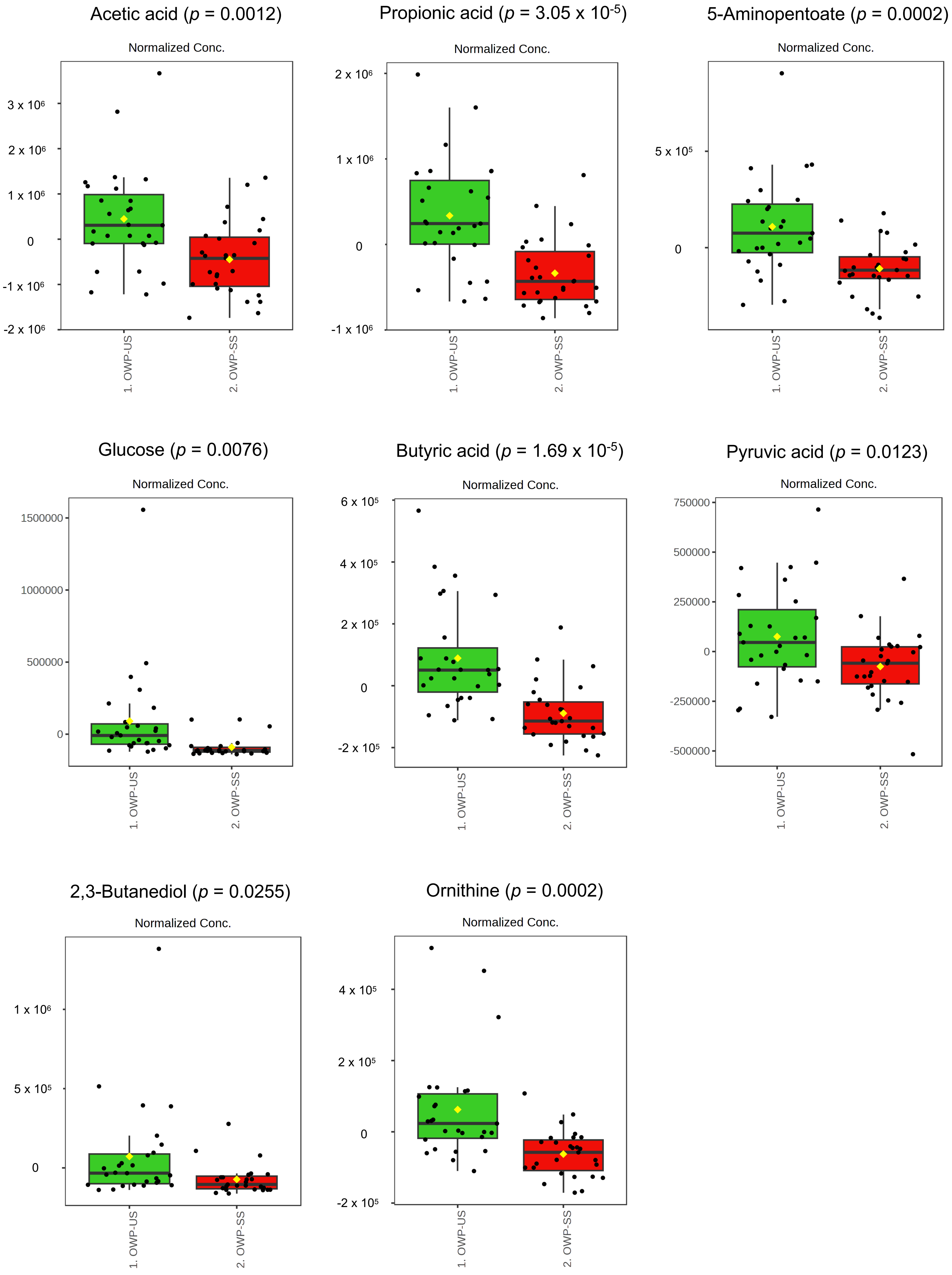
Acetic acid (p = 0.0012)
Normalized Conc.
3 x 10^6
2 x 10^6
1 x 10^6
0
-1 x 10^6
-2 x 10^6
1. OWP-US
2. OWP-SS
Propionic acid (p = 3.05 x 10^-5)
Normalized Conc.
2 x 10^6
1 x 10^6
0
-1 x 10^6
1. OWP-US
2. OWP-SS
5-Aminopentoate (p = 0.0002)
Normalized Conc.
5 x 10^5
0
1. OWP-US
2. OWP-SS
Glucose (p = 0.0076)
Normalized Conc.
1500000
1000000
500000
0
1. OWP-US
2. OWP-SS
Butyric acid (p = 1.69 x 10^-5)
Normalized Conc.
6 x 10^5
4 x 10^5
2 x 10^5
0
-2 x 10^5
1. OWP-US
2. OWP-SS
Pyruvic acid (p = 0.0123)
Normalized Conc.
750000
500000
250000
0
-250000
-500000
1. OWP-US
2. OWP-SS
2,3-Butanediol (p = 0.0255)
Normalized Conc.
1 x 10^6
5 x 10^5
0
1. OWP-US
2. OWP-SS
Ornithine (p = 0.0002)
Normalized Conc.
4 x 10^5
2 x 10^5
0
-2 x 10^5
1. OWP-US
2. OWP-SS

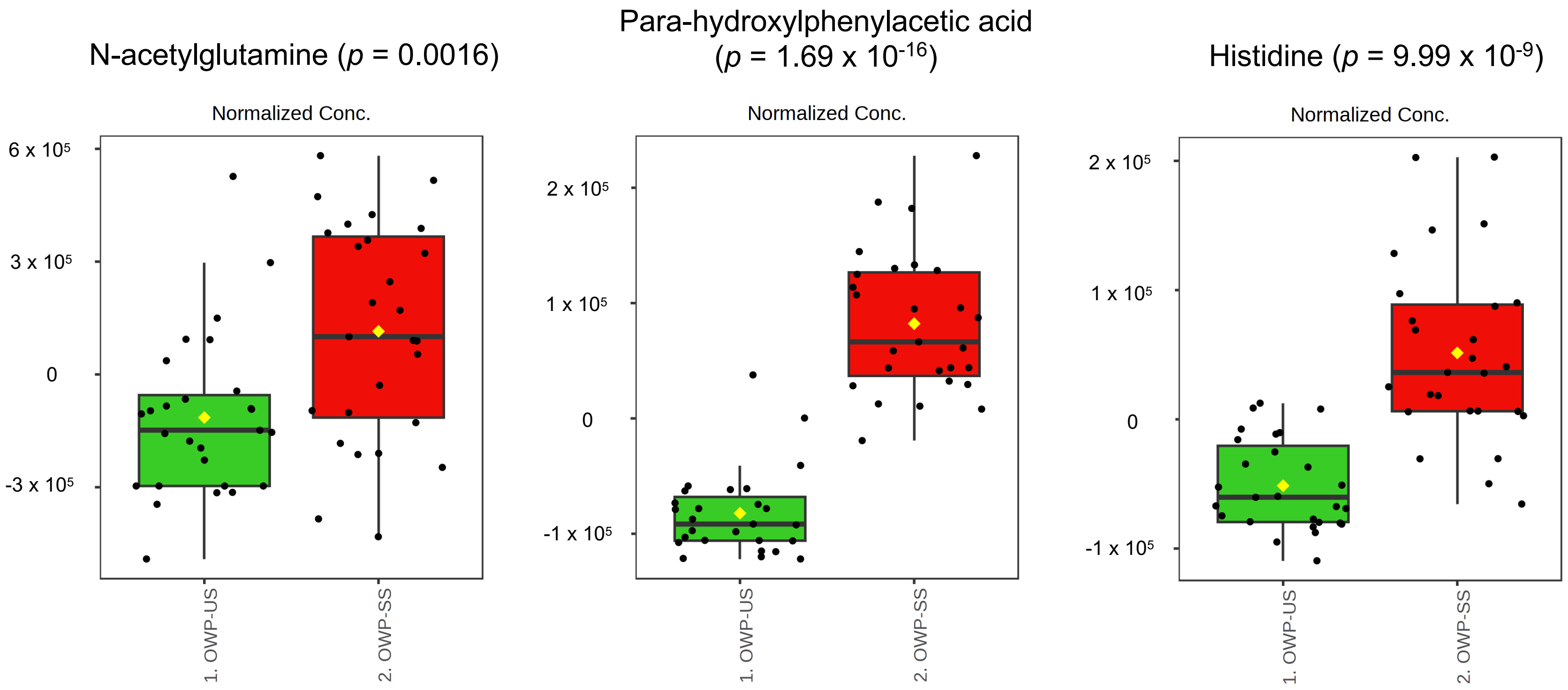
N-acetylglutamine (p = 0.0016)
Para-hydroxylphenylacetic acid (p = 1.69 x 10^{-16})
Histidine (p = 9.99 x 10^{-9})
Normalized Conc.
6 x 10^5
3 x 10^5
0
-3 x 10^5
2 x 10^5
1 x 10^5
-1 x 10^5
1. OWP-US
2. OWP-SS
