# Supplementary material for: Comparison of the Metabolic Profile between Unstimulated and Stimulated Saliva Samples from Pregnant Women with/without Obesity and Periodontitis
Source: J Pers Med. 2023 Jul 11;13(7):1123. doi: 10.3390/jpm13071123 (PMC10381358; doi:10.3390/jpm13071123)
Supplement: Supplementary file 1 [file jpm-13-01123-s001.zip › Supplementary file S4.pdf]

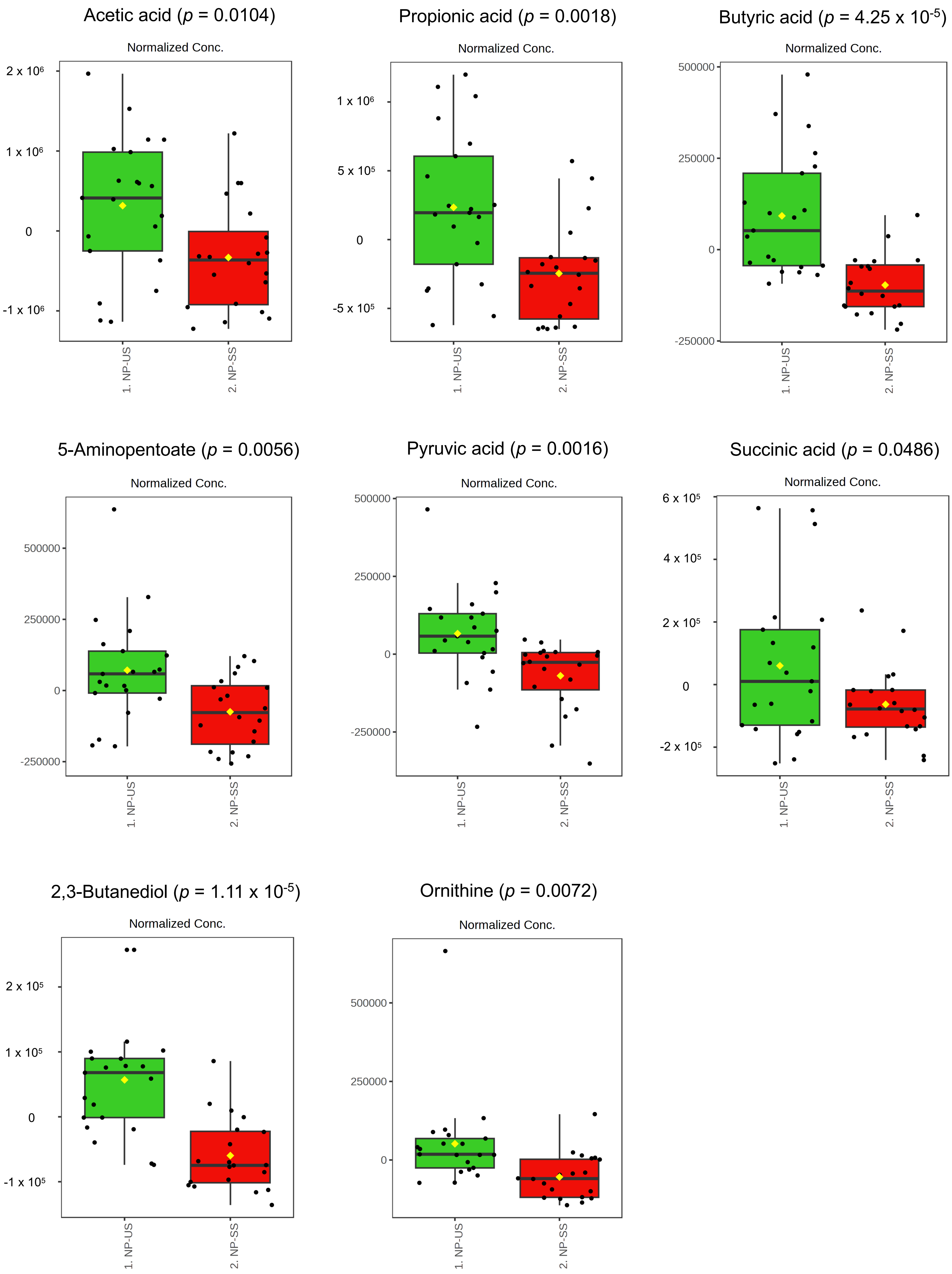
Acetic acid (p = 0.0104)
Normalized Conc.
2 x 10^6
1 x 10^6
0
-1 x 10^6
1. NP-US
2. NP-SS
Propionic acid (p = 0.0018)
Normalized Conc.
1 x 10^6
5 x 10^5
0
-5 x 10^5
1. NP-US
2. NP-SS
Butyric acid (p = 4.25 x 10^-5)
Normalized Conc.
500000
250000
0
-250000
1. NP-US
2. NP-SS
5-Aminopentoate (p = 0.0056)
Normalized Conc.
500000
250000
0
-250000
1. NP-US
2. NP-SS
Pyruvic acid (p = 0.0016)
Normalized Conc.
500000
250000
0
-250000
1. NP-US
2. NP-SS
Succinic acid (p = 0.0486)
Normalized Conc.
6 x 10^5
4 x 10^5
2 x 10^5
0
-2 x 10^5
1. NP-US
2. NP-SS
2,3-Butanediol (p = 1.11 x 10^-5)
Normalized Conc.
2 x 10^5
1 x 10^5
0
-1 x 10^5
1. NP-US
2. NP-SS
Ornithine (p = 0.0072)
Normalized Conc.
500000
250000
0
1. NP-US
2. NP-SS

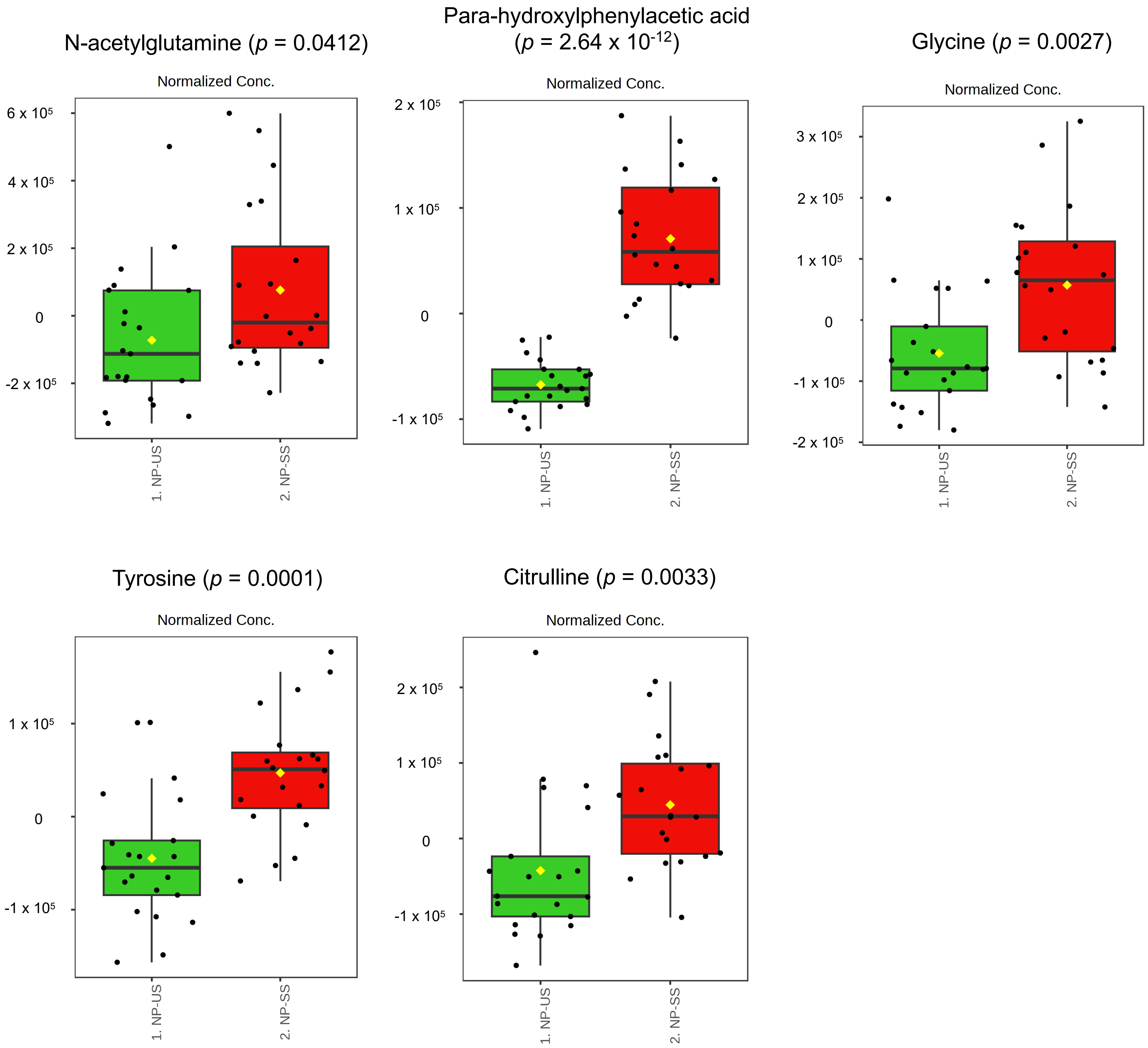

N-acetylglutamine (p = 0.0412)
Normalized Conc.
6 x 10^5
4 x 10^5
2 x 10^5
0
-2 x 10^5
1. NP-US
2. NP-SS
Para-hydroxylphenylacetic acid ($p = 2.64 \times 10^{-12}$)
Normalized Conc.
2 x 10^5
1 x 10^5
0
-1 x 10^5
1. NP-US
2. NP-SS
Glycine (p = 0.0027)
Normalized Conc.
3 x 10^5
2 x 10^5
1 x 10^5
0
-1 x 10^5
-2 x 10^5
1. NP-US
2. NP-SS
Tyrosine (p = 0.0001)
Normalized Conc.
1 x 10^5
0
-1 x 10^5
1. NP-US
2. NP-SS
Citrulline (p = 0.0033)
Normalized Conc.
2 x 10^5
1 x 10^5
0
-1 x 10^5
1. NP-US
2. NP-SS
