# Supplementary material for: Comparison of the Metabolic Profile between Unstimulated and Stimulated Saliva Samples from Pregnant Women with/without Obesity and Periodontitis
Source: J Pers Med. 2023 Jul 11;13(7):1123. doi: 10.3390/jpm13071123 (PMC10381358; doi:10.3390/jpm13071123)
Supplement: Supplementary file 1 [file jpm-13-01123-s001.zip › Supplementary file S5.pdf]

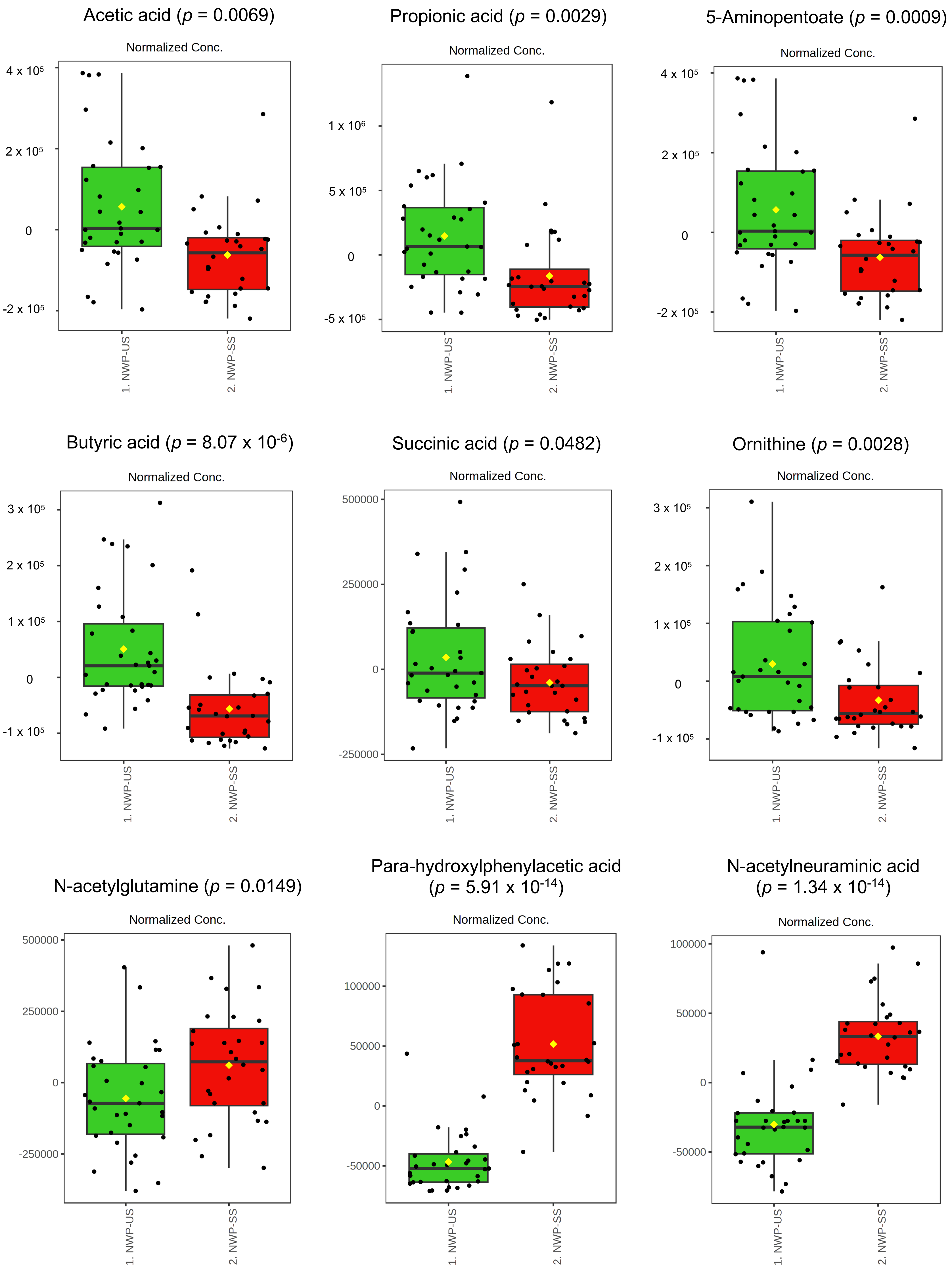
Acetic acid (p = 0.0069)
Normalized Conc.
4 x 10⁵
2 x 10⁵
0
-2 x 10⁵
1. NWP-US
2. NWP-SS
Propionic acid (p = 0.0029)
Normalized Conc.
1 x 10⁶
5 x 10⁵
0
-5 x 10⁵
1. NWP-US
2. NWP-SS
5-Aminopentoate (p = 0.0009)
Normalized Conc.
4 x 10⁵
2 x 10⁵
0
-2 x 10⁵
1. NWP-US
2. NWP-SS
Butyric acid (p = 8.07 x 10⁻⁶)
Normalized Conc.
3 x 10⁵
2 x 10⁵
1 x 10⁵
0
-1 x 10⁵
1. NWP-US
2. NWP-SS
Succinic acid (p = 0.0482)
Normalized Conc.
500000
250000
0
-250000
1. NWP-US
2. NWP-SS
Ornithine (p = 0.0028)
Normalized Conc.
3 x 10⁵
2 x 10⁵
1 x 10⁵
0
-1 x 10⁵
1. NWP-US
2. NWP-SS
N-acetylglutamine (p = 0.0149)
Normalized Conc.
500000
250000
0
-250000
1. NWP-US
2. NWP-SS
Para-hydroxylphenylacetic acid
(p = 5.91 x 10⁻¹⁴)
Normalized Conc.
100000
50000
0
-50000
1. NWP-US
2. NWP-SS
N-acetylneuraminic acid
(p = 1.34 x 10⁻¹⁴)
Normalized Conc.
100000
50000
0
-50000
1. NWP-US
2. NWP-SS

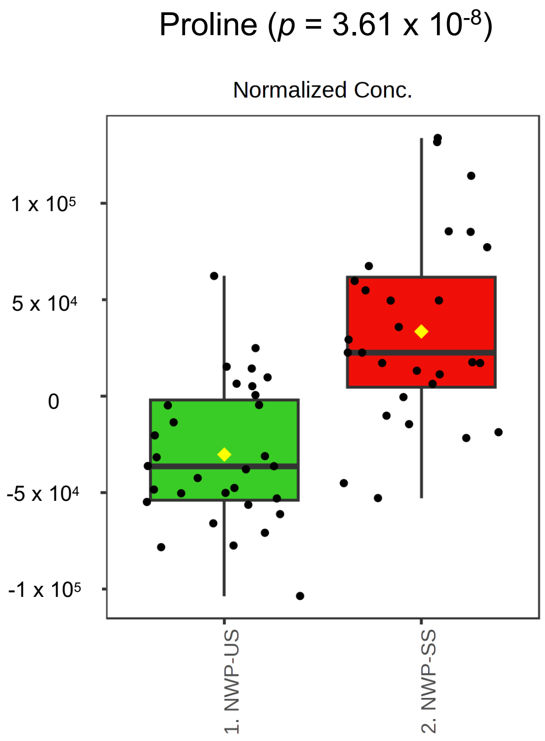

Proline (p = 3.61 x 10-8)
Normalized Conc.
1 x 105
5 x 104
0
-5 x 104
-1 x 105
1. NWP-US
2. NWP-SS
